# Consideration for Hemiballismus in the Differential Diagnosis: A Rare Case of Hyperosmolar Hyperglycemic State

**DOI:** 10.7759/cureus.27416

**Published:** 2022-07-28

**Authors:** Iman Isayli, Nicolas Ulloa, John Childress

**Affiliations:** 1 Emergency Medicine, Graduate Medical Education, Aventura Hospital and Medical Center, Aventura, USA

**Keywords:** nonketotic hyperglycinemia, dka, hyperosmolar hyperglycemic state, hhs, diabetes, glycemic control, focal seizures, uncontrolled diabetes, hemiballismus, diabetes mellitus

## Abstract

We present a case of a 58-year-old male with a past medical history of hypertension and diabetes mellitus presenting to the emergency department with a complaint of abnormal and uncontrollable right arm jerking motions occurring since the afternoon on the day prior to presentation. Arm movements such as these may be consistent with either focal seizures or hemiballismus, a movement disorder classified as a choreiform subtype consisting of involuntary violent movements of an extremity with wide amplitudes. Although oftentimes focal seizures and hemiballismus are associated with neurologic etiologies such as strokes, the second most common cause of hemiballismus appears to be non-ketotic hyperosmolar hyperglycemia. While symptomatic treatment in managing focal seizures and hemiballismus may consist of benzodiazepines and dopamine receptor antagonists, respectively, it is important to treat the underlying cause, which in this case was the non-ketotic hyperosmolar hyperglycemic state associated with this patient’s long-standing history of poorly controlled diabetes mellitus.

## Introduction

Newly diagnosed diabetes mellitus or poorly controlled diabetes mellitus can lead to life-threatening complications, namely, diabetic ketoacidosis (DKA) and hyperglycemic hyperosmolar state (HHS). Both are characterized by hyperglycemia and metabolic derangements due to a lack of insulin. Specifically for our case, it is important to note the wide array of presentations of HHS. Patients in HHS can present with polyuria, polydipsia, seizures, altered mental status, coma, and death [1]. The principles of management in the acute care setting are aggressive intravenous fluid hydration, insulin infusion, and addressing the precipitating cause [1].

## Case presentation

The patient is a 58-year-old male with a past medical history of hypertension, diabetes with a history of the hyperosmolar hyperglycemic state, and chronic kidney disease presenting to the emergency department with abnormal rhythmic, jerking movements of the right upper extremity for one day prior to presentation. He has a documented history of medication nonadherence. Upon questioning his spouse, she mentions he has not been taking his insulin as prescribed. The patient denies any nausea, vomiting, abdominal pain, headache, slurred speech, weakness, numbness, or tingling.

On initial presentation, his vitals were notable for hypertension and tachycardia. Blood pressure was 194/116, heart rate was 104 bpm, pulse oximetry was 98%, respiratory rate was 18, and the temperature was 98.3°F. A physical exam was notable for an adult male who appears his stated age. The patient was in no acute distress. The cardiac, pulmonary, and abdominal examinations were benign. Mucus membranes were dry. He was alert and oriented to person, place, time, and situation. The patient initially had a belt tied around his right forearm and shoulder. After the belt was removed, he developed forceful, rhythmic, jerking movements of the right arm, which caused him some distress.

Being concerned with focal seizures, the patient was given 1 mg of lorazepam IV and loaded with 1 g of levetiracetam IV with an improvement of his symptoms. Laboratory studies were notable for severe hyperglycemia, elevated serum osmolarity, negative ketones, and a bicarbonate level in the lower range of the normal reference value as seen in Table 1. Computed tomography of the brain was negative for any acute findings.

**Table 1:** Pertinent patient lab values BUN: Blood urea nitrogen; mg/dL: Milligrams per deciliter; mOsm/Kg: Milliosmoles per kilogram of water; mmol/L: Millimoles per liter.

Lab Values	Results	References
Serum Glucose	1043 mg/dL	70-100 mg/dL
Serum Osmolarity	336 mOsm/Kg	27,50,399 mOsm/Kg
Serum Ketones	Positive	Negative or undetectable
Serum Sodium	126 mmol/L	135-145 mmol/L
Serum Potassium	4.1 mmol/L	3.5-5.2 mmol/L
Serum Chloride	92 mmol/L	95-110 mmol/L
Serum Bicarbonate	19 mmol/L	19-34 mmol/L
Serum BUN	54 mg/dL	6-22 mg/dL
Serum Creatinine	6.40 mg/dL	0.43-1.13 mg/dL

The patient was then aggressively rehydrated with 3 L of lactated Ringer's solution, and an insulin Novolin drip was started at a rate of 6 mL/h in the emergency department. He was admitted to the intensive care unit (ICU) for HHS with suspected focal seizures. While in the ICU, neurology was consulted who recommended electroencephalogram (EEG) and magnetic resonance imaging (MRI) of the brain. EEG demonstrated no focal abnormalities, epileptogenic discharges, or hemispheric asymmetries. MRI of the brain without contrast was negative for any acute focal lesion, bleeds, or infarcts. This study was only notable for bilateral maxillary and ethmoid sinus disease. The patient had an uncomplicated hospital course and was discharged to home on antiepileptics. Patient follow-up revealed improved adherence to his diabetic regimen, and the patient denied continued abnormal movements of his extremities.

## Discussion

This case is an excellent demonstration of the complications related to diabetes mellitus as well as the wide array of neurologic manifestations that occur with HHS. Patients tend to present with polydipsia, polyuria, altered mental status, coma, and sometimes seizures [1]. HHS is a laboratory diagnosis in which patients must have glucose greater than 600 mg/dl, serum osmolarity greater than 320 mOsm/kg, and negative ketones [1]. Typically associated with type II diabetes, these patients tend to be older and present with a precipitating illness [1]. HHS can also be due to medication nonadherence with delay in seeking medical attention, as seen in our case.

After a review of the literature, it became more apparent that hemiballismus secondary to HHS should be considered in the patient's differential diagnosis. HHS is the second leading cause of hemiballismus, only preceded by cerebrovascular ischemia [2]. Other causes of hemiballismus include central nervous system (CNS) infections, neoplasm, and other neurodegenerative disorders [3]. Hemiballismus is described as a movement disorder classified as a choreiform subtype consisting of involuntary, violent movements of an extremity with wide amplitudes, such as those movements observed in our patient [4].

The pathophysiology is not well understood, but it is theorized that the hyperglycemia and other associated metabolic derangements often seen in HHS lead to hyperviscosity, decreased cerebral blood flood flow, and ischemia, mainly affecting the basal ganglia [5-7]. In some case reports, the CT imaging or MRI imaging of the brain demonstrates areas of hyperdensity in the basal ganglia with the absence of mass effect, edema, or further signs of hemorrhage [7-9]. This region of the brain is responsible for GABAergic (gamma-aminobutyric acid) projection neurons, and thus, ischemia in the basal ganglia (as indicated by the region of hyperdensity) could explain the mechanism of the movement disorder seen in these patients [5-9].

In HHS-associated hemiballismus, symptoms eventually resolve with an improvement of hyperglycemia [10]. These specific patients tend to have a favorable prognosis if they maintain adequate glycemic control [10,11]. Occasionally, medications such as benzodiazepines, neuroleptics, and antiepileptics are also utilized [10]. Patients in HHS also may present with focal seizures. Focal seizures may occur in up to 10%-25% of patients in HHS [5]. In these patients, their focal seizures will oftentimes be refractory to anti-epileptic drugs alone and will instead be responsive to insulin therapy and hydration [12-14]. Our patient received lorazepam, antiepileptics as well as aggressive management of his severe hyperglycemia; therefore, it is difficult to determine which aspect of management led to the resolution of his symptoms.

Nevertheless, both hemiballismus and focal seizures are focal syndromes that are associated with the disruption of GABAergic projection neurons [5-9]. Thus, both diagnoses would be treated similarly by addressing and resolving the patient’s hyperosmolar hyperglycemic state.

## Conclusions

HHS can have a wide array of presentations and neurological complications. It is paramount to recognize and treat this condition promptly. The challenging aspect of our case was its unusual presentation. Patients tend to be encephalopathic or comatose, yet this patient’s sole complaint was the abnormal movement of his upper extremity. Focal seizures and hemiballismus are both possible complications of HHS. Symptoms tend to improve with glycemic control, but other agents may be helpful such as benzodiazepines, neuroleptics, and antiepileptics. The mechanism of hemiballismus in HHS remains poorly understood.
